# Distinguishing pleiotropy from linked QTL between milk production traits and mastitis resistance in Nordic Holstein cattle

**DOI:** 10.1186/s12711-020-00538-6

**Published:** 2020-04-07

**Authors:** Zexi Cai, Magdalena Dusza, Bernt Guldbrandtsen, Mogens Sandø Lund, Goutam Sahana

**Affiliations:** 1 Faculty of Technical Sciences, Center for Quantitative Genetics and Genomics, Aarhus University, 8830 Tjele, Denmark; 2 Department of Animal Sciences, University of Agriculture in Kraków, 30-059 Kraków, Poland

## Abstract

**Background:**

Production and health traits are central in cattle breeding. Advances in next-generation sequencing technologies and genotype imputation have increased the resolution of gene mapping based on genome-wide association studies (GWAS). Thus, numerous candidate genes that affect milk yield, milk composition, and mastitis resistance in dairy cattle are reported in the literature. Effect-bearing variants often affect multiple traits. Because the detection of overlapping quantitative trait loci (QTL) regions from single-trait GWAS is too inaccurate and subjective, multi-trait analysis is a better approach to detect pleiotropic effects of variants in candidate genes. However, large sample sizes are required to achieve sufficient power. Multi-trait meta-analysis is one approach to deal with this problem. Thus, we performed two multi-trait meta-analyses, one for three milk production traits (milk yield, protein yield and fat yield), and one for milk yield and mastitis resistance.

**Results:**

For highly correlated traits, the power to detect pleiotropy was increased by multi-trait meta-analysis compared with the subjective assessment of overlapping of single-trait QTL confidence intervals. Pleiotropic effects of lead single nucleotide polymorphisms (SNPs) that were detected from the multi-trait meta-analysis were confirmed by bivariate association analysis. The previously reported pleiotropic effects of variants within the *DGAT1* and *MGST1* genes on three milk production traits, and pleiotropic effects of variants in *GHR* on milk yield and fat yield were confirmed. Furthermore, our results suggested that variants in *KCTD16*, *KCNK18* and *ENSBTAG00000023629* had pleiotropic effects on milk production traits. For milk yield and mastitis resistance, we identified possible pleiotropic effects of variants in two genes, *GC* and *DGAT1*.

**Conclusions:**

Multi-trait meta-analysis improves our ability to detect pleiotropic interactions between milk production traits and identifies variants with pleiotropic effects on milk production traits and mastitis resistance. In particular, this should contribute to better understand the biological mechanisms that underlie the unfavorable genetic correlation between milk yield and mastitis.

## Background

Holstein is an important cattle breed in the Danish dairy production and much effort has gone in the genetic improvement of its milk production and functional traits. Intense selection for increased milk yield has negative consequences on the udder health of cows [1]. Unfavorable genetic correlations between milk production and clinical mastitis (from 0.21 to 0.55) have been reported [2]. A genetic correlation between two traits could be due to the pleiotropic action of genetic variants or the correlation (i.e., linkage disequilibrium (LD)) between causal variants. The identification of a quantitative trait locus (QTL) that affects simultaneously milk yield and udder health can help reveal some of the genetic basis of the genetic connection between milk production and mastitis resistance. In combination with specific genetic tests, this information can contribute to reduce the unfavorable correlated response on mastitis due to selection that focused on improving milk production traits by differentially weighting variants based on their favorable or unfavorable effects on the two traits.

One application of genome-wide association studies (GWAS) is to detect pleiotropic effects for the QTL identified from single-trait analysis. If a genomic region is significant for two or more traits, it may be due to causal variants that are in LD and affect individual traits (linkage), or that these traits are affected by the same variant (pleiotropy). The number of segregating variants in a population is large, but finite. The proportion of the segregating variants that are associated with the genetic variation of complex traits is unknown. However, traits often appear to be associated with the same or closely-linked variants in the genome [3, 4], which strongly suggests that, at least some of the underlying causal variants, affect several traits. Therefore, the primary aim of this analysis was to determine whether QTL associated with more than one trait were indeed pleiotropic. We used our previous GWAS results (summary statistics) of milk production traits [3] and mastitis resistance [4] to perform a multi-trait meta-analysis for scanning lead SNPs associated with three milk production traits or with milk yield and mastitis resistance for pleiotropy. In combination with a bivariate analysis, we examined the possible pleiotropic nature of the QTL identified.

## Methods

### Animals and phenotypes

We used de-regressed proofs (DRP) based on estimated breeding values (EBV) [5, 6] for milk, fat, protein yields and mastitis resistance (udder health index, which is an index for clinical mastitis from first to third lactation) for about 5000 Nordic Holstein (HOL) bulls. Nordic Cattle Genetic Evaluation (https://www.nordicebv.info/) provided the EBV.

### Genotype and sequence data

We performed an association analysis using imputed whole-genome sequence (WGS) data. All bulls (~ 5000) were genotyped with the BovineSNP50 BeadChip SNP array (54 k) versions 1 or 2 (Illumina Inc., San Diego, CA). Imputation to WGS variants was described earlier by Iso-Touru et al. [7]. Briefly, 54k genotypes were imputed to WGS variants by a 2-step approach. First, using a multi-breed reference of 3383 animals (1222 Holstein (HOL), 1326 Nordic Red cattle (RDC) and 835 Jersey (JER)) that had been genotyped with the Illumina Bovine HD SNP array (Illumina Inc., San Diego, CA), all the animals were imputed to the high-density (HD) level. Next, the imputed HD genotypes were imputed to the WGS level using a multi-breed reference of 1228 animals: 1148 WGS from *Run4* of the 1000 Bull Genomes Project (288 Holstein, 56 Red, and 61 Jersey, as well as 743 individuals from various breeds) [8] and 80 animals from Aarhus University (23 HOL, 30 RDC, and 27 JER). Imputation to HD genotypes was done with the IMPUTE2 v2.3.1 software [9], and imputation to the whole-genome level with the Minimac2 software [10]. The average accuracy (r^2^-values from Minimac2) was 0.85 for across-breed imputation. Imputation accuracy was previously reported by Wu et al. [11].

Before phasing and imputation, we filtered the 54 k and HD genotypes based on an SNP call rate higher than 85% and an animal call rate higher than 90%. The imputed sequence data included 22,751,039 bi-allelic variants. For each breed, SNPs with a minor allele frequency (MAF) lower than 1% or with a highly significant deviation from Hardy–Weinberg proportions (p < 1.0^−6^) were excluded. After quality filtering, 16,503,508 SNPs remained for analysis.

### Single-SNP association analysis for a trait

The GWAS summary statistics were from two previous association analyses [3, 4] and, here, we provide a brief description of the GWAS method used. The genetic relationship matrix (GRM) was estimated using imputed HD genotypes by the GCTA software [12]. We followed the leave-one-chromosome-out approach [13] to build a kinship matrix that was specific to the analysis of each chromosome by leaving out markers on that chromosome to avoid loss of power due to double-counting (fitting the SNP as a fixed effect for testing associations and as a random effect as part of the GRM) [14].

First, we performed a single-SNP GWAS analysis using GCTA [12] for each chromosome. A Bonferroni multiple-testing correction was applied to control for false-positive associations: a SNP was significant if its test probability p-value, $p_{M}$, was less than 0.05/ $M$, where $M$ is the number of SNPs. This corresponds to a trait-wise nominal type 1 error-rate of 5%. Here, the significance threshold value was − log_10_($p_{M} = 8. 5$) with $M \approx 1 5. 3 6$ million SNPs. We identified the lead SNPs for each independent QTL signal on a chromosome by iteratively fitting the allele dosages of the lead SNPs identified in the previous runs as covariate (for details see [3, 4]).

### Genetic variance explained by the identified QTL

We used GCTA [12] to estimate the genetic variance explained by all the identified QTL together for each trait. First, we extracted the genotype for all lead SNPs identified from the GWAS and generated the first GRM. Next, we excluded all SNPs within the 2.5-Mb region around each lead SNP and estimated the second GRM with the remaining SNPs. Finally, we estimated the genetic variance explained by each of these two groups of variants for each trait by fitting two GRM in a linear mixed model.

### Defining a QTL region

QTL regions were defined as continuous regions that include a lead SNP with a − log_10_(p) > 8.50. The start and the end of the QTL region were determined based on the following considerations: (1) the value of 3 was subtracted from the -log_10_(p) value of the lead SNP; (2) from the remaining SNPs, we identified those that were located furthest to the left and right with a− log_10_(p) value no less than 3 units below the − log_10_(p) of the lead SNP of the region; the positions of these SNPs were taken as boundaries of the QTL region, but if they were further than 0.25 Mb (left or right from the lead SNP), then the size of the QTL region was limited by 0.25 Mb.

### Estimation of genetic correlations

We used a linkage disequilibrium score regression approach as implemented in the LDSC software [15] to estimate the genetic correlation between traits using GWAS summary statistics. For polygenic traits, the more a SNP is in LD with other genetic variants, the greater is its chance of being correlated with causal variants, and the higher is its expected association test statistic. Exploiting this relationship allows the estimation of SNP-based heritability when using association test statistics for a single trait or the estimation of SNP-based co-heritability when combining association test statistics from two traits. The LD score of a SNP is the sum of the LD (r^2^) of the SNP with other SNPs and, thus, can be regarded as a measure of the genetic variation that is ‘tagged’ by the SNP. First, we calculated the LD scores for each variant using WGS data of Holstein animals from Run6 in the 1000 Bull Genome Project [8] and of additional Holstein individuals from Aarhus University. Then, GWAS summary statistics from our previous studies [3, 4] were converted to the input format of the LDSC software using the accompanying script *munge_sumstats.py* (part of LDSC software). The reformatted summary statistics were used to calculate genetic correlations between traits.

### Multi-trait meta-analysis

A multi-trait meta-analysis was performed using the approximate multi-trait test statistic described by Bolormaa et al. [16]. Effects of a SNP across all traits were calculated and combined with the genomic correlation matrix between traits to perform a multi-trait $\chi^{2}$ test with a number of degrees of freedom equal to the number of traits. The formula to compute the multi-trait statistic for SNP $i$ was $\chi_{MT,i}^{2} = {\mathbf{t}}_{i}^{'} {\mathbf{V}}^{ - 1} {\mathbf{t}}_{i}$, where ${\mathbf{t}}_{i}$ is a vector of signed $t$ test statistics for the association of lead SNP $i$ with each trait obtained by single trait GWAS, and ${\mathbf{V}}^{ - 1}$ is the inverse of the genomic correlation matrix for all traits. The same Bonferroni-corrected significance threshold as in the single trait association analyses (i.e., − log_10_($p_{M}$) > 8.5) was applied in the multi-trait analyses.

### Single-SNP bivariate association analysis

A single-SNP bivariate association analysis was carried out for each lead SNP from the multi-trait meta-analysis. The bivariate model used for a SNP is as follows

$$\left[ {\begin{array}{*{20}c} {{\mathbf{y}}_{1} } \\ {{\mathbf{y}}_{2} } \\ \end{array} } \right] = \left[ {\begin{array}{*{20}c} {\mu_{1} {\mathbf{1}}} \\ {\mu_{2} {\mathbf{1}}} \\ \end{array} } \right] + \left[ {\begin{array}{*{20}c} {\beta_{1} m} \\ {\beta_{2} m} \\ \end{array} } \right] + \left[ {\begin{array}{*{20}c} {{\mathbf{Z}}_{1} } & {\mathbf{0}} \\ {\mathbf{0}} & {{\mathbf{Z}}_{2} } \\ \end{array} } \right]\left[ {\begin{array}{*{20}c} {{\mathbf{u}}_{1} } \\ {{\mathbf{u}}_{2} } \\ \end{array} } \right] + \left[ {\begin{array}{*{20}c} {{\mathbf{e}}_{1} } \\ {{\mathbf{e}}_{2} } \\ \end{array} } \right],$$

where subscripts $1$ and $2$ indicate traits 1 and 2 in the analysis, ${\mathbf{y}}_{i}$ are the vectors of phenotypes for trait $i$, $\mu_{i}$ is the general mean for trait $i$, ${\mathbf{m}}$ is a vector of genotype doses for the lead SNP, $\beta_{i}$ is the allele substitution effect of the lead SNP for trait $i$, ${\mathbf{Z}}_{i}$ is a design matrix relating phenotypic observations to polygenic effects for trait $i$, $${\mathbf{u}} = \left( {\begin{array}{*{20}c} {{\mathbf{u}}_{1} } \\ {{\mathbf{u}}_{2} } \\ \end{array} } \right)$$ is a vector of the random polygenic effects with a multivariate normal distribution ${\mathbf{u}}\sim N\left( {0,{\mathbf{G}} \otimes {\mathbf{A}}} \right)$, where ${\mathbf{A}}\varvec{ }$ is the additive relationship matrix and ${\mathbf{G}}$ is the polygenic covariance matrix, and $${\mathbf{e}} = \left( {\begin{array}{*{20}c} {{\mathbf{e}}_{1} } \\ {{\mathbf{e}}_{2} } \\ \end{array} } \right)$$ is a vector of mutually independent residual terms with a multivariate normal distribution $${\mathbf{e}}\sim N\left( {0,\left( {\begin{array}{*{20}c} {\sigma_{e1}^{2} } & 0 \\ 0 & {\sigma_{e2}^{2} } \\ \end{array} } \right) \otimes {\mathbf{I}}} \right)$$, where $\sigma_{ei }^{2}$ is the residual variance for trait $i$, and ${\mathbf{I}}$ is an identity matrix of appropriate dimensions. The model was fit by AI-REML using DMU [17].

### Pleiotropy vs. variants in linkage disequilibrium

A bivariate model might help to distinguish between a variant that affects both traits (via different paths), and a variant that has an effect on one trait that is mediated through another trait. In a bivariate model, the effects of SNPs are expected to be significant for both traits in the first scenario, but only for one of the traits in the second scenario. To distinguish between pleiotropic effects and effects of distinct variants in LD, we conducted bivariate analyses (as described above) for the lead SNPs that were detected in the multi-trait meta-analysis. The lead SNPs that showed genome-wide significance for at least one of the traits in the bivariate analyses and a significance p < 1.18e−4 ($p_{N}$=0.05/ $N$, where $N$ is equal to number of traits (i.e. 4) times the number of unique lead SNPs (i.e. 106) identified across all traits) for the other trait were considered to have a pleiotropic effect on both traits.

### Candidate genes underlying the associated genomic regions

Annotations for the lead SNPs for each QTL region from the single-trait analyses and the meta-analysis along with the genes that harbor the lead SNP were determined via the cow (*Bos taurus*) genome assembly UMD3.1 [18]. We used the variant effect predictor (VEP) software (ver. 92.0) [19] to predict the functional consequences of the lead SNPs and identify the closest gene.

## Results

### Single-variant association analysis and genetic correlation

**Fig. 1:**
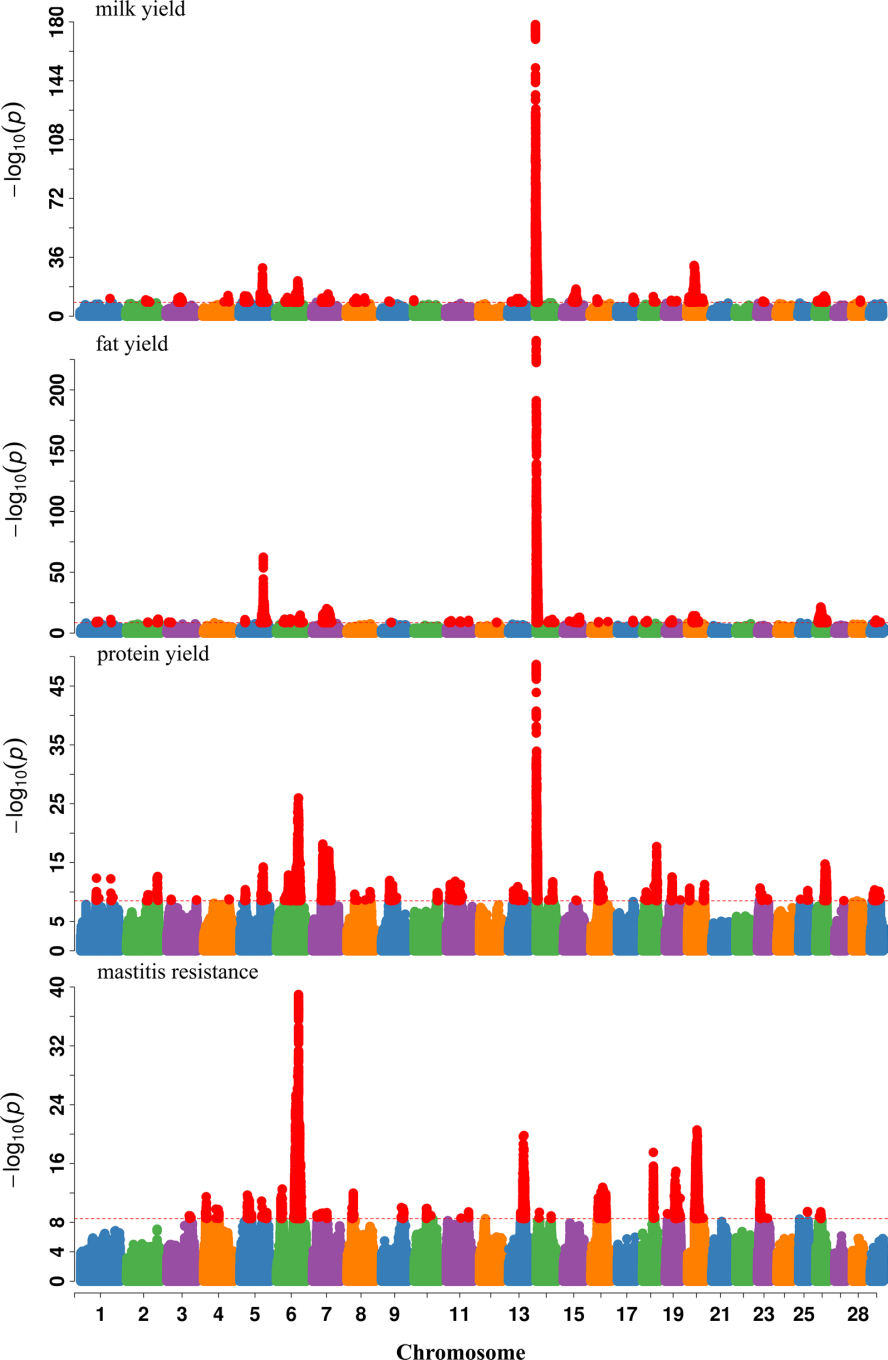
Manhattan plots for single-trait GWAS for fat yield (FY), protein yield (PY), milk yield (MY) and mastitis resistance (MR). Each color corresponds to an autosome. The horizontal red dotted line shows the genome-wide Bonferroni corrected significance threshold [− log10(p) = 8.5]. Base positions refer to the UMD 3.1.1 [18] bovine genome assembly

Previously, we published the results of a GWAS for milk production traits and mastitis resistance [3, 4], which are summarized in Fig. 1 and Table 1. We identified 27 independent association signals on 18 chromosomes for fat yield (FY), 34 association signals on 22 chromosomes for protein yield (PY), 26 association signals on 20 chromosomes for milk yield (MY), and 22 association signals on 18 chromosomes for mastitis resistance (MR). Several QTL detected for different traits were located in close proximity. Table 2 lists the genetic correlations between MY, FY, PY and MR as estimated by LDSC. Moderate to strong genetic correlations between MY, FY and PY were observed but unfavorable genetic correlations between each of the three milk production traits and MR were found, as reported previously [2, 20].

**Table 1:** Summary of the GWAS results for milk production traits and mastitis resistance

Traits	Number of QTL	V (lead SNP)/V (DRP)^a^ (%)
Fat yield	27	28.57
Protein yield	34	16.76
Milk yield	26	21.50
Mastitis resistance	22	14.54

^a^Percentage of genetic variance explained by the identified QTL, V(lead SNP) is the variance explained by the lead SNPs, whereas V(DRP) is the variance of de-regressed breeding values (DRP)

**Table 2:** Genetic correlations between milk yield (MY), fat yield (FY), protein yield (PY) and mastitis resistance (MR) estimated from GWAS summary statistics

Traits	MY	FY	PY	MR
MY		0.40 (0.14)	0.78 (0.03)	− 0.35 (0.04)
FY			0.56 (0.17)	− 0.20 (0.05)
PY				− 0.27 (0.04)

Numbers in parentheses are standard errors of genetic correlation estimates

### Three-trait meta-analysis for fat, protein, and milk yields

We examined the overlap between QTL regions for FY, PY, and MY (Table 3). Some of the overlapping QTL regions did not contain any genes, such as the two regions 20,035,379–20,534,779 bp and 93,703,737–93,762,020 bp on *Bos taurus* autosome (BTA)5, and 2044,412–2049,435 bp on BTA14 (Table 3). In contrast, the QTL intervals on BTA14 and 19 included several overlapping regions that included many genes.

**Table 3:** Overlapping QTL intervals identified based on single-trait GWAS for milk yield (MY), fat yield (FY) and protein yield (PY)

Chr.	Region	Traits	Genes
2	85393563–86241732	FY, MY	*ENSBTAG00000047452*, *GTF3C3*, *CCDC150*, *PGAP1*, *ANKRD44*
5	20035379–20534779	FY, PY	–
5	93703737–93762020	FY, PY, MY	–
5	93762020–94198670	FY,PY	*MGST1, SLC15A5*
5	93698481–93703737	FY, MY	*LMO3*
14	1545264–1583427	FY, PY, MY	bta-mir-2308, *ARHGAP39*
14	1448510–1545264	FY, MY	*ZNF16*, *C14H8orf33*, *ZNF34*, *ZNF7*, *COMMD5*, *RPL8*
14	1549272–2044412	FY, PY, MY	bta-mir-2308, C14H8orf82, *LRRC14*, *RECQL4*, *GPT*, *PPP1R16A*, *KIFC2*, *VPS28*, *SLC52A2*, *TMEM249*, *SCX*, *ENSBTAG00000044406*, *ENSBTAG00000039978*, *HGH1*, *MAF1*, *SHARPIN*, *CYC1*, *GPAA1*, *EXOSC4*, *ENSBTAG00000015040*, *GRINA*, *PARP10*, *LRRC24*, *MFSD3*, *FOXH1*, *CYHR1*, *TONSL*, *SLC39A4*, *CPSF1*, *ADCK5*, *FBXL6*, *SCRT1*, *DGAT1*, *HSF1*, *MROH1*, *WDR97*, *SPATC1*, *ARHGAP39*, *BOP1*, *OPLAH*
14	2044412–2049435	FY, MY	–
14	67731848–68231920	FY, MY	*KCNS2*, *STK3*, *ENSBTAG00000046739*
16	31268349–31746789	FY, MY	*ENSBTAG00000044816*, *H3F3C*, *ENSBTAG00000021109*, *ENSBTAG00000042363*, *SCCPDH*, *TFB2M*, *SMYD3*, *CNST*
19	27156952–27692965	FY, PY, MY	*PSMB6*, *GLTPD2*, *VMO1*, *CXCL16*, *MED11*, *ARRB2*, bta-mir-2338, *ALOX15*, *ALOX12E*, ENSBTAG00000047925, *RNASEK*, C19H17orf49, bta-mir-497, bta-mir-195, *BCL6B*, *SLC16A13*, *CLEC10A*, *ASGR1*, *ENSBTAG00000042630*, *ACADVL*, bta-mir-324, *PHF23*, *GABARAP*, *ELP5*, *CLDN7*, *SLC2A4*, *EIF5A*, *GPS2*, *NEURL4*, *ENSBTAG00000045892*, *ACAP1*, *KCTD11*, *PLD2*, *TM4SF5*, *ZMYND15*, *PELP1*, *ALOX12*, *SLC16A11*, *ASGR2*, *DLG4*, *DVL2*, *CTDNEP1*, *YBX2*, *MINK1*
19	26625240–27156952	FY, PY	*ENSBTAG00000025126*, *MIS12*, *C1QBP*, *RPAIN*, bta-mir-199c, *ENSBTAG00000013906*, *SPAG7*, *ENO3*, *ENSBTAG00000004913*, *SLC25A11*, *GP1BA*, *CHRNE*, C19H17orf107, *NLRP1*, *DERL2*, *DHX33*, *SCIMP*, *ZFP3*, *KIF1C*, *INCA1*, *CAMTA2*, *PFN1*, *NUP88*, *RABEP1*, *MINK1*
19	27692965–27773922	FY, MY	*PLSCR3*, *TMEM256*, *NLGN2*, *SPEM1*, *TMEM102*, *CHRNB1*, *TNK1*, *FGF11*, *ZBTB4*

**Fig. 2:**
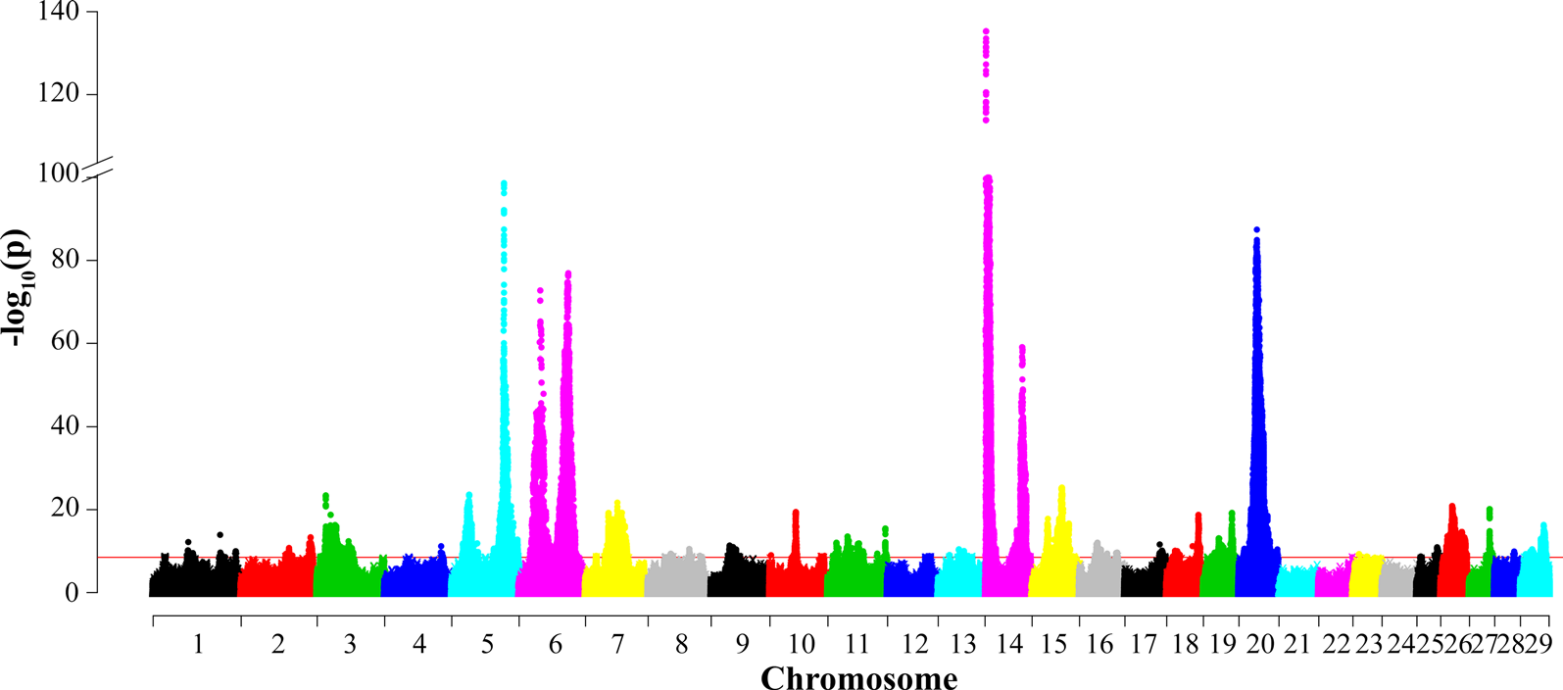
Manhattan plot of the multi-trait meta-analysis for milk, fat and protein yields. The red horizontal line indicates the genome-wide significance level [− log10(p) = 8.5]. Base positions refer to the UMD 3.1.1 [18] bovine genome assembly

We performed a multi-trait meta-analysis for FY, PY and MY to examine if the lead SNP affected multiple milk production traits (Fig. 2). In total, we identified 59 association signals across 27 chromosomes (Table 4). One peak on BTA5, two peaks on BTA6, two peaks on BTA14 and one peak on BTA20 showed strong association signals in the meta-analysis. The strongest signal was located on BTA14 and resulted from the well-known and previously described SNPs BTA14:1802,265 (rs109234250) and BTA14:1802,266 (rs109326954) in the *DGAT1* gene [21, 22]. These two SNPs were also the lead SNPs in the single-trait analyses for FY and MY with a –log_10_(p) value greater than 240 and 178, respectively. These two SNPs were in complete LD and had identical p values for both traits. The single-trait analysis for PY did not identify these two causal variants as the ‘lead’ SNP. Instead, the strongest associated SNP in this region for PY was SNP BTA14:1835,440 (rs208567981) with –log_10_(p) = 48.66. This variant was located within the *BOP1* gene, but very close to *DGAT1* [3], whereas the two causal variants (BTA14:1802,265 and BTA14:1802,266) had –log_10_(p) = 47.99 in the analysis for PY [3].

**Table 4:** The lead SNP and its nearest genes in the multi-trait meta-analysis of milk yield (MY), fat yield (FY) and protein yield (PY)

Chr	Lead SNP (bp)	rsid	− log_10_(p)	Nearest gene (distance)	Annotation	Single trait significant^a^
1	63177947	rs42409534	12.19	*ENSBTAG00000046854* (7850,66 bp)	Intergenic	FY, PY
1	71250238	rs210007164	9.51	*TFRC* (9830 bp)	Intergenic	FY
1	120470021	rs109519395	13.92	*AGTR1* (67,012 bp)	Intergenic	FY, MY, PY
1	148567669	rs108957710	9.97	*ENSBTAG00000046447*	Upstream	Novel
2	86095020	rs110457337	10.76	*ANKRD44*	Intron	FY, PY
2	124837669	–	13.34	*PTPRU*	Intron	FY, PY
3	15459025	rs132797166	23.45	*GBA*	Intron	Novel
4	101547644	–	11.18	*CHRM2*	Upstream	MY
5	31335325	rs447206924	23.61	*LALBA* (12,536 bp)	Intergenic	MY
5	93945991	rs208248675	126.98	*MGST1*	Intron	FY, MY
6	38031954	rs454966322	72.75	*ABCG2* (1371 bp)	Downstream	FY, MY
6	88442678	rs211069111	76.92	*SLC4A4*	Intron	PY
7	57287990	–	21.70	*KCTD16*	Intron	FY, MY, PY
8	40627140	rs381636155	9.38	bta-mir-2471 (64,540 bp)	Intergenic	MY
8	73877814	rs381189656	10.50	*ENSBTAG00000010829* (171 bp)	Upstream	MY, PY
8	93065787	rs211543235	8.88	*GRIN3A*	Intron	PY
9	33179984	rs211241852	11.33	*SLC35F1* (83,834 bp)	intergenic	MY, PY
10	1989907	rs109274615	9.01	*ENSBTAG00000047622* (109,990 bp)	Intergenic	MY
10	46591115	rs211044009	19.41	*USP3* (20,126 bp)	Intergenic	Novel
10	93933304	–	8.73	*SEL1L*	Intron	PY
11	15339847	rs110446044	12.01	*TTC27*	Intron	FY, PY
11	35270918	rs109956079	13.52	*ENSBTAG00000027786* (241,298 bp)	Intergenic	PY
11	55692712	rs208208268	11.86	*CTNNA2*	Intron	FY, PY
11	63203084	rs110286816	9.96	*ENSBTAG00000046117* (87,338 bp)	intergenic	Novel
11	88771449	rs109730673	9.41	*ENSBTAG00000047976* (150,709 bp)	Intergenic	FY
11	103301805	rs110788821	15.43	*PAEP*	Intron	Novel
12	75309869	rs43128997	8.78	*ENSBTAG00000026070* (148,028 bp)	Intergenic	Novel
13	20194953	rs41602070	9.06	*ITGB1* (53,992 bp)	Intergenic	PY
13	37208793	rs385962673	10.43	*MKX* (10,635 bp)	Intergenic	MY, PY
13	46391099	rs42442665	10.03	*ADARB2*	Intron	MY
13	60701113	rs108986421	8.61	*RSPO4* (9143 bp)	Intergenic	PY
14	1802265	rs109234250	705.26	*DGAT1*	Missense	FY, MY, PY
14	1802266	rs109326954	705.26	*DGAT1*	Missense	FY, MY, PY
14	66649826	rs445616049	59.08	*MGC148714* (2105 bp)	Downstream	Novel
15	28357864	rs208325660	17.79	*RNF214*	Missense	Novel
15	53640417	rs380720492	25.33	*ARHGEF17*	Synonymous	MY
15	66103726	rs41775109	16.64	*EHF* (72,762 bp)	Intergenic	FY
16	32262983	rs384531912	12.03	*SMYD3*	Intron	PY
16	49798794	rs380930173	9.40	*AJAP1* (33,340 bp)	intergenic	PY
16	67736535	rs385935762	9.64	*SWT1*	intron	Novel
17	62543160	rs211016475	11.60	*TBX5*	Intron	FY
18	15058306	rs211386971	10.04	*VPS35*	Intron	PY
18	46583596	rs110398208	11.19	*UPK1A* (662 bp)	Upstream	MY, PY
18	57064406	rs433130247	18.74	*MYBPC2*	Intron	PY
19	27522927	rs210334173	13.09	*ASGR1* (6170 bp)	Intergenic	FY, PY
19	51383847	rs136067046	19.23	*FASN* (1075 bp)	Upstream	Novel
20	9608408	rs519502268	10.57	*CARTPT* (183,253 bp)	Intergenic	MY, PY
20	31909478	rs385640152	112.79	*GHR*	Missense	FY, MY
20	69006609	rs134612291	10.41	*IRX1* (466,030 bp)	Intergenic	PY
22	60087443	rs462776871	8.51	*EEFSEC*	Intron	Novel
23	10974968	rs136158431	9.29	*FGD2* (56,619 bp)	Intergenic	PY
23	25076739	rs210864958	8.70	*GCM*	Downstream	MY
25	6984796	rs110991040	8.71	*RBFOX1* (298,008 bp)	Intergenic	Novel
25	36299420	rs210351939	10.92	*ACHE* (1112 bp)	Upstream	Novel
26	20527926	rs453802222	20.87	*ENSBTAG00000023629* (175 bp)	Downstream	FY
26	37869471	rs458256022	14.61	*KCNK18*	Synonymous	FY, MY, PY
27	36212352	rs208675276	20.10	*GPAT4*	5_prime_UTR	Novel
28	34972377	rs137526033	9.90	*ZMIZ1* (60,078 bp)	Intergenic	MY
29	21075705	rs382642281	10.42	*ENSBTAG00000000853* (161,330 bp)	Intergenic	FY, PY
29	41825511	–	16.32	*STX5*	Intron	Novel

The distance in base pairs from the nearest gene is in brackets

^a^Novel hit from multi-trait meta-analysis, not identified by any of the single trait analysis

The multi-trait meta-analysis can help to deal with accuracy of the single-trait analysis. The causal variant known in *GHR* (F279Y) [23] was the lead SNP on BTA20 from the meta-analysis (Table 4). However, in the single-trait analysis for FY and MY, the causative variant did not emerge as the lead SNP [3]. In addition, on BTA5, we detected the second lead SNP at BTA5:31,335,325 (rs447206924, Table 4). The nearest gene to this lead SNP is *LALBA*, which encodes α-lactalbumin. The multi-trait meta-analysis helped to pinpoint this known causal gene whereas both the single-trait analysis for MY [3] and the overlapping QTL regions between milk production traits (Table 3) failed to do so.

The lead SNPs detected in the meta-analysis were either lead SNPs from the single-trait analyses (18 lead SNPs) or those the most closely located to the lead SNPs identified by the single-trait analyses (Table 4). Moreover, the meta-analysis identified 16 additional association signals that were not genome-wide significant in the single-trait analyses (Table 4). We searched the mammalian phenotype database [24] to verify the candidate genes that were suggested by the multi-trait meta-analysis. In addition to *DGAT1*, *MGST1*, *ABCG2* and *GHR*, we identified one more gene with biological support, *GPAT4*. The term in the mammalian phenotype database showed that certain alleles of the *GPAT4* gene cause “abnormal milk composition” in mouse [25].

### Two-trait meta-analysis for milk yield and mastitis resistance

Two overlapping QTL regions for MY and MR were detected in this study on BTA5 and 6 (Table 5). The QTL region on BTA5 harbors several genes and that on BTA6 (88.6 to 89.1 Mb) harbors the *GC* and *NPFFR2* genes, which have been reported to be associated with clinical mastitis in cows [26].

**Table 5:** Genes located within the overlapping QTL regions detected in the single-trait GWAS between milk yield and mastitis resistance

Chr	Region	Genes
5	30202453–31258920	bta-mir-2425, *DNAJC22*, *TROAP*, *PRPH*, *TUBA1C*, *TUBA1A*, *DHH*, *RHEBL1*, bta-mir-2426, *PRKAG1*, *DDN*, *WNT1*, *WNT10B*, *FKBP11*, *CCDC65*, *CACNB3*, *ENSBTAG00000047525*, *BCDIN3D*, *NCKAP5L*, *TMBIM6*, *FMNL3*, *PRPF40B*, *MCRS1*, *KCNH3*, *C1QL4*, *ENSBTAG00000037775*, *TUBA1B*, *LMBR1L*, *RND1*, *DDX23*, *ADCY6*, *CCNT1*, *SPATS2*, *KMT2D*, *FAM186B*
6	88598011–89097608	*NPFFR2*, *GC*

**Fig. 3:**
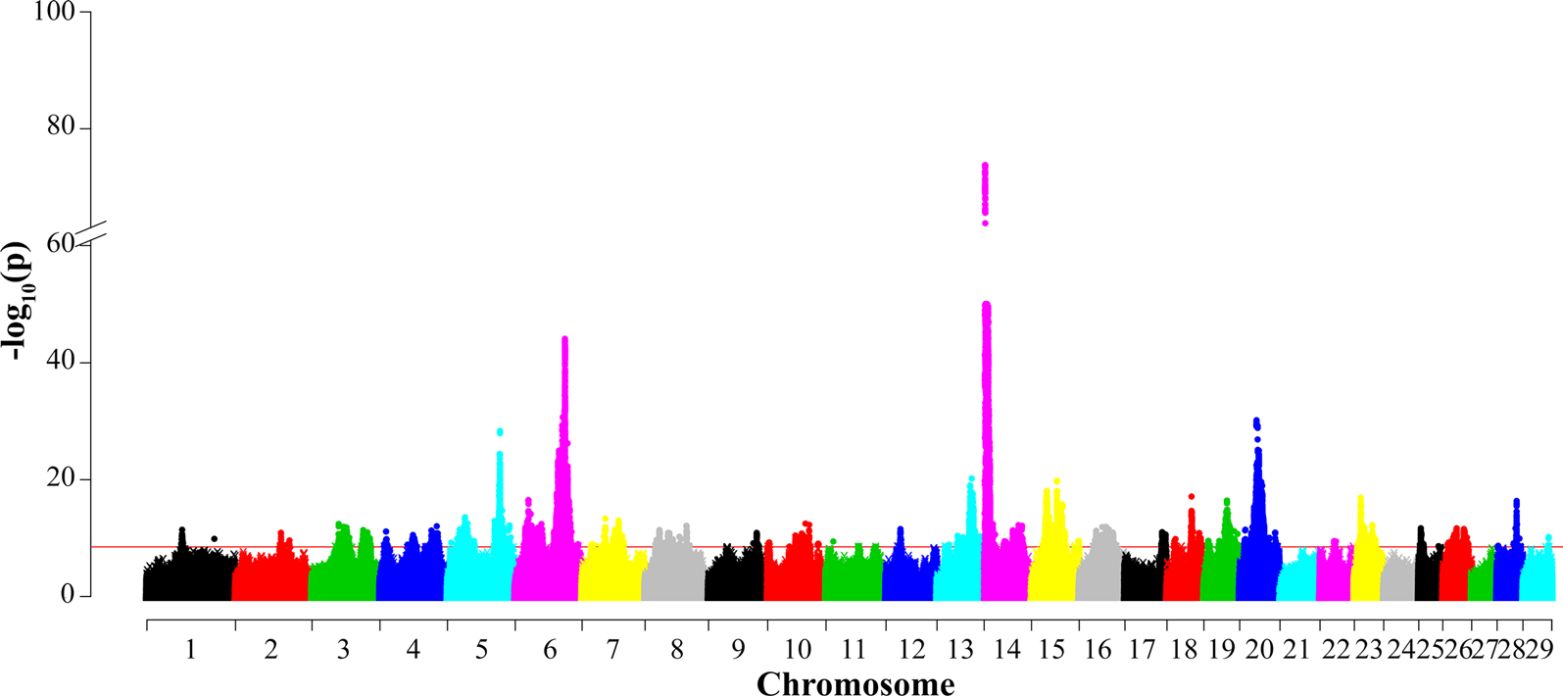
Manhattan plot for the multi-trait analysis of milk yield and mastitis resistance of Nordic Holstein cattle. The red horizontal line indicates the genome-wide significance level [− log_10_(p) = 8.5]. Base positions refer to the UMD 3.1.1 [18] bovine genome assembly

The most significant signal in the meta-analysis was located on BTA14:1793,616 (Table 6 and Fig. 3) and 1735 bp upstream of *DGAT1*. We believe that this signal was caused by the two known causal mutations in *DGAT1*. However, this lead SNP was significant only in the single-trait analysis for MY, but not for MR (Table 6). The second strongest association signal was located on BTA6:88,729,872 in the *GC* gene. The third strongest association signal was on BTA5:93,953,487, close to *MGST1* but this lead SNP was significant only in the single-trait analysis for MY, and not in that for MR (Table 6).

**Table 6:** The lead SNP and its nearest genes in the multi-trait meta-analysis of milk yield (MY) and mastitis resistance (MR)

Chr	Lead SNP (bp)	rsid	− log_10_(p)	Nearest gene (distance)	Annotation	Single-trait significant^a^
1	62876378	–	11.43	*ENSBTAG00000046854* (483,497 bp)	Intergenic	Novel
2	81179721	rs110121625	10.92	*TMEFF2*	Intron	MY
2	96376739	–	8.92	*RF00001* (13,015 bp)	Intergenic	Novel
3	47643148	rs378327566	12.42	*PTBP2* (732,492 bp)	Intergenic	MY
3	91848036	–	11.21	*USP24*	Intron	Novel
4	10938389	rs211526380	11.12	*TFPI2* (97,090 bp)	Intergenic	MR
4	59012300	rs136891032	10.53	*ENSBTAG00000020620* (287,400 bp)	Intergenic	Novel
4	101547644	–	12.03	*CHRM2* (4968 bp)	Upstream	MY
5	31352419	–	13.58	*LALBA* (2537 bp)	Downstream	MR, MY
5	93953487	rs210234664	28.36	*MGST1* (3325 bp)	Upstream	MY
6	23474516	rs109255104	16.50	*MANBA*	Intron	MR
6	88729872	rs109803407	44.04	*GC*	Intron	MR, MY
7	18085661	rs133896398	8.98	*FBN3* (9071 bp)	Intergenic	Novel
7	41607423	rs208385619	13.33	*ENSBTAG00000039706* (16,819 bp)	Intergenic	MR, MY
7	65370850	rs109644389	12.99	*GLRA1* (258,214 bp)	Intergenic	MY
8	25684799	–	11.38	*ADAMTSL1*	Intron	MR
8	41148951	–	10.90	*ENSBTAG00000014467* (197,503 bp)	Intergenic	MY
8	61253437	rs43552270	10.23	*MELK* (22,888 bp)	Intergenic	Novel
8	73877814	rs381189656	12.11	*ENSBTAG00000010829* (171 bp)	Upstream	MY
9	86108587	rs209751747	10.90	*SAMD5* (87,968 bp)	Intergenic	MR
10	2752616	rs209970861	9.26	*YTHDC2* (318,445 bp)	Intergenic	Novel
10	39050124	–	8.56	*ENSBTAG00000004692* (137,772 bp)	Intergenic	Novel
10	49633928	rs43587750	10.44	*RORA*	Intron	Novel
10	67479321	rs109694327	12.49	*SAMD4A*	Intron	MR
10	74236917	rs380306966	12.27	*SNAPC1* (58,791 bp)	Intergenic	Novel
10	90272296	rs381454149	9.06	*ADCK1* (137,647 bp)	Intergenic	Novel
11	13025259	–	9.43	*DYSF*	Intron	Novel
11	58017848	rs380589113	8.64	*LRRTM4* (415,739 bp)	Intergenic	Novel
11	88743727	rs380133715	8.53	*ID2* (156,561 bp)	Intergenic	MR
12	25763482	rs210185748	11.55	*NBEA* (29,504 bp)	Intergenic	Novel
13	21477118	rs135125951	8.87	*RF00026* (80,513 bp)	Intergenic	Novel
13	36822330	rs379020207	10.39	*MPP7*	Intron	MY
13	46391099	rs42442665	10.13	*ADARB2*	intron	MY
13	62017506	rs211080099	20.20	*PDRG1* (2545 bp)	Upstream	MR
13	76532010	rs42057265	10.31	*ZMYND8*	Intron	Novel
14	1793616	rs384957047	183.77	*DGAT1* (1735 bp)	Upstream	MY
14	36478894	rs43757971	9.46	*XKR9* (1379 bp)	Upstream	Novel
14	61344981	rs42484846	12.22	*ZFPM2*	Intron	MR
15	27475189	rs208648732	18.08	*RF00285* (140,736 bp)	Intergenic	Novel
15	45384070	rs43100874	19.84	*ENSBTAG00000048176* (4269 bp)	Upstream	MY
15	84666672	rs382250433	9.54	*MS4A13* (102 bp)	Upstream	Novel
16	30309951	rs384258494	11.26	*STUM*	Intron	MR
16	47836093	rs207941573	11.92	*ACOT7*	Intron	MR
16	59908679	rs451830006	10.94	*BRINP2* (45,408 bp)	Intergenic	MR
17	66530413	rs209525123	11.00	*CORO1C*	Intron	MY
18	13625299	rs209154036	9.53	*ZNF469* (115,323 bp)	Intergenic	Novel
18	43909571	rs464881101	17.12	*ENSBTAG00000004994* (17,651 bp)	Intergenic	MR
18	57501622	–	10.88	*KLK14* (2325 bp)	upstream	Novel
19	7941510	rs209798151	9.51	*TRIM25*	Intron	Novel
19	27442452	rs483221509	9.52	bta-mir-497 (689 bp)	Upstream	MY
19	41169414	rs134338592	16.40	*WIPF2*	Intron	MR
20	10123208	rs207633790	11.45	*GTF2H2* (4035 bp)	Downstream	MY
20	29996719	–	30.18	*MRPS30* (75,496 bp)	Intergenic	MY
20	63369153	rs133899283	10.93	*FAM173B* (241,983 bp)	Intergenic	MY
22	25185357	rs110721487	9.50	*CNTN6*	Intron	Novel
23	11294868	–	16.92	*CMTR1* (8638 bp)	Intergenic	MR
23	32139475	rs477621057	12.23	*CARMIL1*	Intron	Novel
25	3655364	rs379765871	11.68	*CDIP1*	Intron	Novel
25	35354412	rs383829107	8.62	*CUX1*	Intron	MR
26	24938054	rs460832137	11.68	*SFR1* (7550 bp)	Intergenic	MY
26	37716420	rs381336935	11.55	*SHTN1* (26,088 bp)	Intergenic	MY
28	1921500	rs383708617	8.69	*RF00001* (26,734 bp)	Intergenic	Novel
28	34972377	rs137526033	16.36	*ZMIZ1* (60,078 bp)	Intergenic	MY
29	45895253	rs209161829	10.19	*POLD4* (4347 bp)	Downstream	Novel

The distance in base pairs from the nearest gene is in brackets

^a^Novel hit from multi-trait meta-analysis, not identified by single trait analysis

Seventeen of the 64 lead SNPs from the meta-analysis were also lead SNPs for either MY or MR. Most of the remaining lead SNPs were close to the lead SNP in the single-trait analysis [3, 4]. In addition to *DGAT1* and *LALBA*, we found one more candidate gene, *ZFPM2*, with biological support in the mammalian phenotype database [24]. *LALBA* encodes one of the major milk protein, α-lactalbumin. Both *LALBA* and *ZFPM2* are related to the term “abnormal mammary gland morphology”.

### Pleiotropy vs. closely linked variants

To examine if there was evidence for pleiotropic effects of the associated variants, we conducted bivariate analyses for the lead SNPs detected in the multi-trait meta-analysis. The lead SNPs that were genome-wide significant for at least one of the traits from the bivariate analyses are in Table 7. We concluded that a SNP might have pleiotropic effects if it also showed significance (p < 1.18e−4) for the second trait.

**Table 7:** Results of the bivariate analyses with genome-wide significance for at least one trait

Chr	BP	rsid	Trait1	Trait2	P1	P2	Indication of pleiotropy^a^
1	120470021	rs109519395	MY	FY	4.36E−06	7.66E−11	Yes
5	93945991	rs208248675	MY	FY	3.58E−21	1.33E−38	Yes
7	57287990	rs380779883	MY	FY	4.47E−12	1.63E−17	Yes
14	1802265	rs109234250	MY	FY	3.97E−124	6.43E−200	Yes
14	1802266	rs109326954	MY	FY	3.88E−124	6.69E−200	Yes
15	66103726	rs41775109	MY	FY	0.67E−01	1.85E−09	No
20	31909478	rs385640152	MY	FY	2.65E−13	4.06E−08	Yes
26	20527926	rs453802222	MY	FY	6.44E−08	8.67E−16	Yes
26	37869471	rs458256022	MY	FY	2.33E−09	4.55E−08	Yes
1	63177947	rs42409534	MY	PY	5.55E−07	4.94E−11	Yes
1	120470021	rs109519395	MY	PY	2.57E−06	2.12E−10	Yes
5	93945991	rs208248675	MY	PY	1.90E−21	1.82E−06	Yes
6	88442678	rs211069111	MY	PY	2.09E−01	1.10E−13	No
7	57287990	rs380779883	MY	PY	2.05E−12	9.62E−19	Yes
14	1802265	rs109234250	MY	PY	2.31E−125	1.30E−28	Yes
14	1802266	rs109326954	MY	PY	2.26E−125	1.28E−28	Yes
18	57064406	rs433130247	MY	PY	5.44E−06	1.36E−11	Yes
20	31909478	rs385640152	MY	PY	3.02E−13	1.30E−04	No
26	37869471	rs458256022	MY	PY	2.63E−09	3.75E−11	Yes
1	63177947	rs42409534	FY	PY	2.94E−08	3.06E−11	Yes
1	120470021	rs109519395	FY	PY	7.12E−11	2.62E−10	Yes
5	93945991	rs208248675	FY	PY	1.43E−38	1.08E−06	Yes
6	88442678	rs211069111	FY	PY	4.24E−01	1.37E−13	No
7	57287990	rs380779883	FY	PY	1.52E−17	2.56E−18	Yes
14	1802265	rs109234250	FY	PY	4.65E−201	3.93E−29	Yes
14	1802266	rs109326954	FY	PY	4.85E−201	3.88E−29	Yes
15	66103726	rs41775109	FY	PY	1.59E−09	6.91E−01	No
18	57064406	rs433130247	FY	PY	5.93E−01	1.10E−11	No
26	20527926	rs453802222	FY	PY	1.49E−15	1.85E−06	Yes
26	37869471	rs458256022	FY	PY	5.38E−08	3.29E−11	Yes
5	93953487	rs210234664	MY	MR	1.07E−21	1.60E−03	No
6	88729872	rs109803407	MY	MR	5.65E−14	5.04E−33	Yes
11	88743727	rs380133715	MY	MR	1.33E−01	7.37E−10	No
13	62017506	rs211080099	MY	MR	8.25E−01	4.38E−15	No
14	1793616	rs384957047	MY	MR	1.61E−123	8.06E−06	Yes
14	1802667	–	MY	MR	3.41E−123	8.16E−06	Yes
14	1804647	rs109162116	MY	MR	1.97E−123	8.15E−06	Yes
14	1805963	rs211282745	MY	MR	1.89E−123	8.14E−06	Yes
14	1808145	rs135258919	MY	MR	1.97E−123	8.33E−06	Yes
14	1817975	rs135805021	MY	MR	1.97E−123	9.08E−06	Yes
14	1818125	rs383356863	MY	MR	1.96E−123	9.09E−06	Yes
14	1819475	rs208211113	MY	MR	1.89E−123	9.23E−06	Yes
14	1825125	rs208113678	MY	MR	2.58E−123	1.05E−05	Yes
16	47836093	rs207941573	MY	MR	1.19E−01	5.69E−11	No
16	59908679	rs451830006	MY	MR	4.85E−02	2.93E−10	No
18	43909571	rs464881101	MY	MR	4.84E−01	8.09E−11	No
20	29996719	rs43116343	MY	MR	6.08E−13	2.41E−02	No
26	3771642	–	MY	MR	1.24E−10	4.07E−02	No

^a^SNPs showing genome-wide significance for at least one of the traits and significance (p < 1.18e-4) for the second trait

For MY and FY, as expected, we found that the two consecutive missense mutations in *DGAT1* had pleiotropic effects. In addition, we found six other QTL with evidence of pleiotropic effects. On BTA1, we detected the SNP BTA1:120,470,021 with pleiotropic effects on MY and FY. This SNP is located in an intergenic region, close to *AGTR1* (they are 67,012 bp apart). The lead SNPs, BTA5:93,945,991 and BTA7:57,287,990, were each located in an intron of *MGST1* and *KCTD16*, respectively (Table 5). On BTA20, we found that the lead SNP BTA20:31,909,478 located in the *GHR* gene had pleiotropic effects on MY and FY, as previously described [23]. On BTA26, there were two SNPS, BTA26:20,527,926 and BTA26:37,869,471 that were located respectively near *ENSBTAG00000023629* and in *KCNK18* (synonymous variant).

In addition to *DGAT1* and *MGST1*, variants in *KCTD16* and *KCNK18* and an intergenic variant BTA1:120,470,021 were associated with MY and PY. Since these SNPs were also associated with MY and FY, this was the indication that the above-mentioned four genes and one SNP have pleiotropic effects on MY, FY and PY. Meanwhile, BTA1:63,177,947 also showed possible pleiotropic effects for MY, FY and PY, located in an intergenic region close to the gene *ENSBTAG00000046854*.

Apart from the variants in *DGAT1,* only one SNP had significant effects on both MY and MR, i.e. BTA6:88,729,872 (Table 7), which is located in an intron of the *GC* gene.

## Discussion

### Overlapping QTL for three milk production traits

The bivariate analyses showed that the QTL for three milk production traits detected in the single-trait analyses and located on BTA5, 14, 20, and 26 might have pleiotropic effects. The univariate analysis identified overlapping QTL regions for all three milk production traits MY, FY and PY on BTA5, 14, 19, 18, and 26, for MY and FY on BTA2, 5, 14, 16, and 19, and for FY and PY on BTA5 and 19. However, without a joint analysis of two traits, it is not possible to determine whether the causal variants in the overlapping regions are the same ones or not.

BTA14 has been widely explored for genes and QTL related to economically important traits (e.g., [27, 28]), including MY, FY and PY. Recently, Nayeri et al. [29] reported that the region between 1.4 and 2.9 Mb on BTA14 was significantly associated with milk, fat and protein production, and with protein and fat deviation in Canadian Holstein cattle. Our findings support their conclusion that this region on BTA14 is strongly associated with milk production traits.

Segregation of QTL that affect milk production traits on BTA5 has already been reported [30, 31]. Based on an association analysis of a large outbred population, Littlejohn et al. [32] reported that a region on BTA5 at 93.9 Mb had pleiotropic effects on milk protein, fat, and lactose yield, milk volume and milk protein and lactose percentage. A 50-kbp interval that contained 632 variants was centered on the SNP with the most significant p value (g.93945738*C *> *T*) in the *MGST1* gene. The *C* allele associated with high milk fat percentage was also associated with increased FY and protein percentage and decreased PY and milk volume. Kemper et al. [33] obtained similar results for the same region with impacts on a subset of the same milk composition traits. These results are consistent with our study that revealed that the QTL on BTA5 at 93.9 Mb had pleiotropic effects on FY, MY and PY (Table 3).

### Overlapping QTL between milk yield and mastitis resistance

The univariate analysis identified two overlapping QTL regions (30.2–31.3 Mb on BTA5 and 88.6–89.1 Mb on BTA6) for MY and MR. However, the bivariate analysis showed that only the QTL on BTA6 was significantly associated with both traits. As shown in Table 3, effects for these traits had opposite directions—an unfavorable effect on MY and a favorable effect on MR. An unfavorable genetic correlation between milk production and clinical mastitis has been reported [20, 34] and the 88.6-89.1 Mb region on BTA6 contributes to this correlation. The QTL for mastitis resistance on BTA6 in this region is consistent with previous reports. In Nordic Holstein cattle, the region most significantly associated with mastitis was on BTA6 at 88.97 Mb [26]. The same region was also associated with mastitis in Nordic Red Cattle, but not in Danish Jersey Cattle [26]. This region includes two genes, *GC* and *NPFFR2* that encode the vitamin D-binding protein precursor (88,695,940 to 88,739,180 bp) and the neuropeptide FF receptor 2 (89,052,210 to 89,059,348 bp), respectively, which can be involved in mastitis.

Sodeland et al. [35] identified a QTL for clinical mastitis on BTA6 in Norwegian Red Cattle with the most significant SNP, BTA-119376, being located at 90,670,190 bp. Klungland et al. [36] also reported a QTL for clinical mastitis on BTA6 and Ogorevc et al. [37] showed that BTA6 harbors several QTL for mastitis resistance. Moreover, the results by Nielsen et al. [38] point to a region on BTA6 near 90 Mb (containing the cluster of casein genes that encode around 80% of the proteins in cow milk) that is associated with milk production traits and mastitis in Norwegian Red cattle.

### Estimation of genetic correlations using GWAS summary statistics

In this study, we estimated the genetic correlations based on GWAS summary statistics using LDSC regression [15]. There are several advantages for using this approach in cattle: (1) LDSC can estimate a genetic correlation based on GWAS summary statistics, which bypasses the limitation of sharing primary data that are the property of industrial partners; and (2) the genetic parameter estimates obtained by using LDSC in human populations are close to the estimates available from quantitative genetic analyses from previous reports. LDSC regression was first applied on human data [15]. LDSC functions well with the LD structure of the human genome. However, the LD structure in cattle is quite different, i.e. LD is much more extensive in cattle than in humans [39]. Using a linear animal test-day model, Hinrichs et al. estimated genetic correlations of 0.29, 0.30 and 0.34 between mastitis resistance and MY, FY, and PY, respectively [40]. In Norwegian Red cattle, a genetic correlation of 0.25 was reported between clinical mastitis and PY [41], whereas in Danish Holstein cattle, it was equal to 0.33 [42]. In another study on Norwegian cattle, Simianer et al. [43] estimated a genetic correlation of 0.472 between mastitis and MY. In our study, we estimated genetic correlations ranging from moderate to high between the milk production traits. Likewise, Hoekstra et al. [44] reported genetic correlations of 0.39 between MY and FY, 0.86 between MY and PY, and 0.56 between FY and PY in Dutch Black and White cows. Another study from the UK Holstein found genetic correlations of 0.69 between MY and FY, 0.88 between MY and PY, and 0.80 between FY and PY [45]. Based on the comparison of our estimates (MY and FY: 0.40, MY and PY: 0.78, FY and PY: 0.56) with those from these previous studies, we conclude that the LDSC approach with summary statistics from GWAS is reliable for the estimation of genetic parameters in cattle.

### The most significant genes (candidate genes)

#### *DGAT1*

In our study, the QTL around 1.6 and 2.1 Mb on BTA14 had the strongest association with milk production traits (MY, PY and FY). The previously reported two missense SNPs (rs109326954 at 1802,266 bp and rs109234250 at 1802,265 bp) resulting in an amino acid change (K232A) were among the top associated variants in the QTL interval on BTA14. However, these two causal variants were not the lead SNPs for MY and PY in the single-trait association study. Imperfect imputation was mentioned as one possible reason by Iso-Touru et al. [7], who obtained similar results (the causal variant at 1802,266 bp not being the most significantly associated SNP) in Nordic Red Cattle. Both the multi-trait meta-analysis and the bivariate analysis indicated these two SNPs as the top associated variants (Tables 4 and 7). This was consistent with previously reported results on the contribution of these *DGAT1* polymorphisms to variation in milk production traits in cattle [21, 22]. The bivariate analysis confirmed the pleiotropic effect of *DGAT1* on FY, PY and MY. In addition, we detected pleiotropic effects of *DGAT1* on MR, which was also reported previously [46].

#### *MGST1*

Raven et al. [47] identified a highly significant QTL on BTA5 at 85–110 Mb for milk production traits, where one of the lead SNPs was located within 3000 bp from *MGST1*. Previously, a GWAS in Nordic Red Cattle [7] reported a region associated with FY around 93,945,694 bp on BTA5 and *MGST1* was proposed as candidate gene. Another study [48] found a QTL for MY in the same region i.e. between 92.1 and 93 Mb on BTA5. Although MGST1 is known to bind fatty acids directly, this activity appears to be related to its role as a detoxification enzyme [49], thus the mechanism that would explain an association with milk lipid synthesis/secretion on MY remains unknown. In our study, we observed pleiotropic effects of this QTL on FY and MY.

#### Novel candidate genes

Several genes showed large pleiotropic effects on multiple milk production traits. For a few other genes identified in our study, data in the mammalian phenotype database [24] provided strong support for a possible biological effect on the traits analyzed. For example, a mutation in *GPAT4* is responsible for “abnormal milk composition” in mouse. *ZFPM2* is related to the term “abnormal mammary gland morphology”. In the bivariate analysis, we found that *KCTD16*, which is associated with residual feed intake in pigs and meat quality in cattle [48], had pleiotropic effects on FY, PY and MY. Finally, *KCNK18* showed pleiotropic effects on PY and MY but no obvious biological mechanism linking *KCNK18* to milk production traits was found in the literature.

## Conclusions

In this study, we performed a multi-trait meta-analysis and detected several SNPs that affect both milk production traits and mastitis resistance in dairy cattle, which shows the high power of this approach to detect potential pleiotropy effects compared with the subjective assessment of overlapping single-trait QTL regions. Further confirmation of the lead SNPs from the multi-trait meta-analysis shortened the list of those with possible pleiotropic effects. Bivariate analysis can indicate the pleiotropic effect of a variant. We observed that *DGAT1* and *MGST1* had pleiotropic effects on milk production traits, and *GC* had pleiotropic effects on MY and MR. In addition, our results suggest that *KCTD16* and *KCNK18* might have pleiotropic effects on all three milk production traits analyzed. Our findings add to the knowledge about the genetic determination of milk production traits and mastitis resistance in cattle.

## Data Availability

Genome assembly data were taken from publicly available sources. The assembly UMD_3.1.1 is available for download from NCBI. Part of the whole-genome sequencing data from the 1000 Bull Genomes Project are publically available at NCBI using SRA no. SRP039339 and for the rest, the Board of the 1000 Bull Genome Consortium should be contacted. All annotation information was obtained from a publicly available source (http://www.ensembl.org). Whole-genome sequences from Aarhus University and individual SNP genotype data are available only upon agreement with the breeding organization and should be requested directly from the authors.

## References

[1] Oltenacu PA, Broom DM (2010). The impact of genetic selection for increased milk yield on the welfare of dairy cow. Anim Welfare..

[2] Heringstad B, Chang YM, Gianola D, Klemetsdal G (2005). Genetic association between susceptibility to clinical mastitis and protein yield in norwegian dairy cattle. J Dairy Sci.

[3] Cai Z, Guldbrandtsen B, Lund MS, Sahana G (2019). Dissecting closely linked association signals in combination with the mammalian phenotype database can identify candidate genes in dairy cattle. BMC Genet.

[4] Cai Z, Guldbrandtsen B, Lund MS, Sahana G (2018). Prioritizing candidate genes post-GWAS using multiple sources of data for mastitis resistance in dairy cattle. BMC Genomics..

[5] Goddard M (1985). A method of comparing sires evaluated in different countries. Livest Prod Sci..

[6] Schaeffer LR (1985). Model for international evaluation of dairy sires. Livest Prod Sci..

[7] Iso-Touru T, Sahana G, Guldbrandtsen B, Lund MS, Vilkki J (2016). Genome-wide association analysis of milk yield traits in Nordic Red Cattle using imputed whole genome sequence variants. BMC Genet.

[8] Daetwyler HD, Capitan A, Pausch H, Stothard P, van Binsbergen R, Brondum RF (2014). Whole-genome sequencing of 234 bulls facilitates mapping of monogenic and complex traits in cattle. Nat Genet.

[9] Howie B, Marchini J, Stephens M (2011). Genotype imputation with thousands of genomes. G3 (Bethesda).

[10] Fuchsberger C, Abecasis GR, Hinds DA (2015). Minimac2: faster genotype imputation. Bioinformatics.

[11] Wu X, Guldbrandtsen B, Lund MS, Sahana G (2016). Association analysis for feet and legs disorders with whole-genome sequence variants in 3 dairy cattle breeds. J Dairy Sci.

[12] Yang J, Lee SH, Goddard ME, Visscher PM (2011). GCTA: a tool for genome-wide complex trait analysis. Am J Hum Genet.

[13] Yang J, Zaitlen NA, Goddard ME, Visscher PM, Price AL (2014). Advantages and pitfalls in the application of mixed-model association methods. Nat Genet.

[14] Listgarten J, Lippert C, Kadie CM, Davidson RI, Eskin E, Heckerman D (2012). Improved linear mixed models for genome-wide association studies. Nat Methods.

[15] Bulik-Sullivan BK, Loh PR, Finucane HK, Ripke S, Yang J, Schizophrenia Working Group of the Psychiatric Genomics Consortium (2015). LD Score regression distinguishes confounding from polygenicity in genome-wide association studies. Nat Genet.

[16] Bolormaa S, Pryce JE, Reverter A, Zhang Y, Barendse W, Kemper K (2014). A multi-trait, meta-analysis for detecting pleiotropic polymorphisms for stature, fatness and reproduction in beef cattle. PLoS Genet.

[17] Madsen P, Jensen J, Labouriau R, Christensen OF, Sahana G. DMU—a package for analyzing multivariate mixed models in quantitative genetics and genomics. In Proceedings of the 10th World Congress on Genetics Applied to Livestock Production: 17–22 August 2014; Vancouver.

[18] Zimin AV, Delcher AL, Florea L, Kelley DR, Schatz MC, Puiu D (2009). A whole-genome assembly of the domestic cow, Bostaurus. Genome Biol.

[19] Flicek P, Ahmed I, Amode MR, Barrell D, Beal K, Brent S (2013). Ensembl 2013. Nucleic Acids Res.

[20] Lund MS, Jensen J, Petersen PH (1999). Estimation of genetic and phenotypic parameters for clinical mastitis somatic cell production deviance, and protein yield in dairy cattle using Gibbs sampling. J Dairy Sci.

[21] Grisart B, Coppieters W, Farnir F, Karim L, Ford C, Berzi P (2002). Positional candidate cloning of a QTL in dairy cattle: identification of a missense mutation in the bovine *DGAT1* gene with major effect on milk yield and composition. Genome Res.

[22] Grisart B, Farnir F, Karim L, Cambisano N, Kim JJ, Kvasz A (2004). Genetic and functional confirmation of the causality of the DGAT1 K232A quantitative trait nucleotide in affecting milk yield and composition. Proc Natl Acad Sci USA.

[23] Rahmatalla SA, Muller U, Strucken EM, Reissmann M, Brockmann GA (2011). The F279Y polymorphism of the *GHR* gene and its relation to milk production and somatic cell score in German Holstein dairy cattle. J Appl Genet..

[24] Bult CJ, Eppig JT, Kadin JA, Richardson JE, JA Blake, Mouse Genome Database G (2008). The Mouse Genome Database (MGD): mouse biology and model systems. Nucleic Acids Res.

[25] Beigneux AP, Vergnes L, Qiao X, Quatela S, Davis R, Watkins SM (2006). *Agpat6*—a novel lipid biosynthetic gene required for triacylglycerol production in mammary epithelium. J Lipid Res.

[26] Sahana G, Guldbrandtsen B, Thomsen B, Holm LE, Panitz F, Brondum RF (2014). Genome-wide association study using high-density single nucleotide polymorphism arrays and whole-genome sequences for clinical mastitis traits in dairy cattle. J Dairy Sci.

[27] Ashwell MS, Van Tassell CP, Sonstegard TS (2001). A genome scan to identify quantitative trait loci affecting economically important traits in a US Holstein population. J Dairy Sci.

[28] Wibowo TA, Gaskins CT, Newberry RC, Thorgaard GH, Michal JJ, Jiang Z (2008). Genome assembly anchored QTL map of bovine chromosome 14. Int J Biol Sci..

[29] Nayeri S, Sargolzaei M, Abo-Ismail MK, May N, Miller SP, Schenkel F (2016). Genome-wide association for milk production and female fertility traits in Canadian dairy Holstein cattle. BMC Genet.

[30] Bennewitz J, Reinsch N, Grohs C, Leveziel H, Malafosse A, Thomsen H (2003). Combined analysis of data from two granddaughter designs: a simple strategy for QTL confirmation and increasing experimental power in dairy cattle. Genet Sel Evol..

[31] Khatkar MS, Thomson PC, Tammen I, Raadsma HW (2004). Quantitative trait loci mapping in dairy cattle: review and meta-analysis. Genet Sel Evol..

[32] Littlejohn MD, Tiplady K, Fink TA, Lehnert K, Lopdell T, Johnson T (2016). Sequence-based association analysis reveals an MGST1 eQTL with pleiotropic effects on bovine milk composition. Sci Rep..

[33] Kemper KE, Reich CM, Bowman PJ, Vander Jagt CJ, Chamberlain AJ, Mason BA (2015). Improved precision of QTL mapping using a nonlinear Bayesian method in a multi-breed population leads to greater accuracy of across-breed genomic predictions. Genet Sel Evol..

[34] Heringstad B, Chang YM, Gianola D, Klemetsdal G (2005). Genetic analysis of clinical mastitis, milk fever, ketosis, and retained placenta in three lactations of Norwegian red cows. J Dairy Sci.

[35] Sodeland M, Kent M, Olsen H, Opsal M, Svendsen M, Sehested E (2011). Quantitative trait loci for clinical mastitis on chromosomes 2, 6, 14 and 20 in Norwegian Red cattle. Anim Genet.

[36] Klungland H, Sabry A, Heringstad B, Olsen HG, Gomez-Raya L, Vage DI (2001). Quantitative trait loci affecting clinical mastitis and somatic cell count in dairy cattle. Mamm Genome.

[37] Ogorevc J, Kunej T, Razpet A, Dovc P (2009). Database of cattle candidate genes and genetic markers for milk production and mastitis. Anim Genet.

[38] Nilsen H, Olsen H, Hayes B, Nome T, Sehested E, Svendsen M (2009). Characterization of a QTL region affecting clinical mastitis and protein yield on BTA6. Anim Genet.

[39] Gibbs RA, Taylor JF, Van Tassell CP, Barendse W, Eversole KA, Bovine HapMap Consortium (2009). Genome-wide survey of SNP variation uncovers the genetic structure of cattle breeds. Science.

[40] Hinrichs D, Stamer E, Junge W, Kalm E (2005). Genetic analyses of mastitis data using animal threshold models and genetic correlation with production traits. J Dairy Sci.

[41] Heringstad B, Klemetsdal G, Ruane J (1999). Clinical mastitis in Norwegian cattle: frequency, variance components, and genetic correlation with protein yield. J Dairy Sci.

[42] Hansen M, Lund MS, Sørensen MK, Christensen LG (2002). Genetic parameters of dairy character, protein yield, clinical mastitis, and other diseases in the Danish Holstein cattle. J Dairy Sci.

[43] Simianer H, Solbu H, Schaeffer L (1991). Estimated genetic correlations between disease and yield traits in dairy cattle. J Dairy Sci.

[44] Hoekstra J, van der Lugt AW, van der Werf JHJ, Ouweltjes W (1994). Genetic and phenotypic parameters for milk production and fertility traits in upgraded dairy cattle. Livest Prod Sci..

[45] Kadarmideen HN, Thompson R, Coffey MP, Kossaibati MA (2003). Genetic parameters and evaluations from single- and multiple-trait analysis of dairy cow fertility and milk production. Livest Prod Sci..

[46] Manga I, Říha H (2011). The *DGAT1* gene K232A mutation is associated with milk fat content, milk yield and milk somatic cell count in cattle. Arch Anim Breed..

[47] Raven LA, Cocks BG, Hayes BJ (2014). Multibreed genome wide association can improve precision of mapping causative variants underlying milk production in dairy cattle. BMC Genomics..

[48] Mai M, Sahana G, Christiansen F, Guldbrandtsen B (2010). A genome-wide association study for milk production traits in Danish Jersey cattle using a 50 K single nucleotide polymorphism chip. J Anim Sci.

[49] Iida A, Saito S, Sekine A, Harigae S, Osawa S, Mishima C (2001). Catalog of 46 single-nucleotide polymorphisms (SNPs) in the *microsomal glutathione S*-*transferase 1* (*MGST1*) gene. J Hum Genet.

